# Effect of medicinal plant type and concentration on physicochemical, antioxidant, antimicrobial, and sensorial properties of kombucha

**DOI:** 10.1002/fsn3.873

**Published:** 2018-10-25

**Authors:** Hossein Shahbazi, Hadi Hashemi Gahruie, Mohammad‐Taghi Golmakani, Mohammad H. Eskandari, Matin Movahedi

**Affiliations:** ^1^ Department of Food Science and Technology School of Agriculture Shiraz University Shiraz Iran; ^2^ Department of Horticultural Science School of Agriculture Shiraz University Shiraz Iran

**Keywords:** antimicrobial activity, antioxidant activity, kombucha, medicinal plant

## Abstract

The aim of this study was to evaluate the effects of adding various medicinal plants to kombucha medium and to analyze the changes that occur to its physicochemical, antimicrobial, and sensorial properties. In the first part, measurements were made to determine IC_50_ value, total phenolic content, total flavonoid content, minimum inhibitory concentration, pH, organic acids, and sensorial properties of kombucha that contained cinnamon, cardamom, or Shirazi thyme. Results showed that kombucha samples containing cinnamon exhibited higher antioxidant and antimicrobial activities, more organic acids, and better sensorial scores. In the second part, properties of kombucha containing 25%–100% concentrations of cinnamon were evaluated. The result showed that by increasing the cinnamon concentration, certain increases were observed in the amounts of organic acids and in the magnitudes of antioxidants and antimicrobial activities. In conclusion, antioxidant and antimicrobial activities of kombucha can be increased by adding medicinal plants, especially at higher concentrations.

## 1 INTRODUCTION

Kombucha or tea fungus is a functional beverage originating from Asia and is continuing to gain growing popularity around the world (Jayabalan, Malini, Sathishkumar, Swaminathan, & Yun, 2010). It is a symbiotic culture of acetic acid bacteria (such as *Acetobacter xylinum*,* Acetobacter xylinoides*, and *Bacterium gluconicum*) and yeasts (such as *Schizosaccharomyces pombe*,* Saccharomyces ludwigii*,* Zygosaccharomyces rouxii*,* Candida* spp., and *Pichia* spp.) which are grown in sugared tea, statically at ambient temperatures for more than 2 weeks (Sun, Li, & Chen, 2015). The functional properties of kombucha tea such as its antioxidant and antimicrobial activities are attributed to its polyphenols (i.e., epicatechin, epicatechin‐3‐gallate, epigallocatechin, and epigallocatechin‐3‐gallate), gluconic acid, glucuronic acid, lactic acid, vitamins, amino acids, antibiotics, and a combination of micronutrients produced during fermentation (Nguyen, Nguyen, Nguyen, & Le, 2015; Vijayaraghavan et al., 2000). Natural antioxidants are commonly found in the kombucha tea and may potentially provide extremely useful functions, such as antitumor properties, anticarcinogenic, and inhibitory mutagenic reactions—applications that are clinically valuable. Phenolic compounds are among the most abundant of ingredients found in the kombucha (Jayabalan, Subathradevi, Marimuthu, Sathishkumar, & Swaminathan, 2008).

Medicinal plants are one of the most important sources of phenols and flavonoid components (Hashemi Gahruie, Eskandari, Mesbahi, & Hanifpour, 2015; Hashemi Gahruie & Niakousari, 2017; Pourmorad, Hosseinimehr, & Shahabimajd, 2006). Some spices are known to contain components that possess antioxidant and antimicrobial activities, such as the presence of eugenol in cloves, carvacrol in Shirazi thyme (Hashemi Gahruie, Hosseini, et al., 2017), and cinnamic aldehyde in cinnamon.

The aim of this study was to evaluate the effects of adding various concentrations of medicinal plants to the kombucha, so as to monitor the changes that occur to the physicochemical, antimicrobial, and sensorial properties of the kombucha tea.

## 2 MATERIALS AND METHODS

### 2.1 Materials

The Folin–Ciocalteu reagent and the 2,2‐diphenyl‐1‐picrylhydrazyl (DPPH) were purchased from Sigma‐Aldrich Company (St. Louis, MO, USA). All other chemicals and the Mueller Hinton Broth were of analytical grade, purchased from Merck Company (Darmstadt, Germany).

*Staphylococcus aureus* ATCC 29213, *Bacillus cereus* ATCC 10987*, Escherichia coli* O157:H7 ATCC 43895, and *Salmonella typhimurium* ATCC 19430 were provided kindly form microbiology laboratory of food science and technology department (Shiraz University, Shiraz, Iran).

### 2.2 Preparation of kombucha tea

Tea fungus was purchased from a local pharmacy in Shiraz, Iran. The sucrose solution at 6.5% (w/v) was autoclaved for 15 min at 121°C. According to preliminary experiments, appropriate kombucha tea formulations were established (0.7% green tea, 6.5% sucrose solution, and the starter culture). Initially, the sucrose solution (6.5%) and the green tea and medicinal plant were allowed to be brewed for 15 min and then were filtered through a sieve. The tea then cooled down to reach 25°C, and the kombucha tea broth was inoculated by 4% of freshly grown tea fungus. The mixture was covered with tissue paper towels. Fermentation was carried out in an incubator at 28°C.

Three medicinal plants, namely, cinnamon (CIN), cardamom (CAR), and Shirazi thyme (SHT) were applied to the kombucha medium to evaluate the effects of medicinal plants on the physicochemical, antioxidant, and sensorial properties of final kombucha samples, and then the kombucha was mixed with green tea at a ratio of 50:50 (0.35%, w/w: 0.35%, w/w) (which yields the flavored kombucha). This was compared with pure green tea which served as the control. The physicochemical, antioxidant, antimicrobial, and sensorial properties of kombucha samples were analyzed for 16 days at 4‐day intervals.

Based on the results of sensorial evaluations and the analysis of antimicrobial and antioxidant activities, cinnamon was initially chosen as the best medicinal plant for the production of flavored kombucha. The kombucha was prepared according to the method described previously. Four different ratios of cinnamon:green tea were prepared (100:0, 75:25, 50:50, and 25:75). The effect of cinnamon concentration on physicochemical, antioxidant, and antimicrobial activities of kombucha samples was analyzed by the method described in the first part which lasted for 16 days by intervals of 4 days.

### 2.3 Determination of organic acids by HPLC

Samples with volumes of 5 ml were passed through membrane filters (0.45 μM). Ten μl of filtrate sample was injected into the HPLC system (VARIAN, Pro Star, USA). The Aminex^®^ HPX‐87H ion exclusion column was used for the analysis. The mobile phase was 0.01 N H_2_SO_4_ (pH 2.27). The flow rate and column temperature were maintained at 0.7 ml/min and 28°C, respectively. Detection was carried out at 210 nm with a Diode Array Detector (DAD 3262). The resolution peaks were recorded on the HPLC chart according to the retention times of standards. The concentrations of organic acids were quantified based on standard curves (Jayabalan, Marimuthu, & Swaminathan, 2007).

### 2.4 Determination of pH

The pH of the fermented kombucha tea was measured with an electronic pH‐meter (Suntex TS‐1, Taiwan) (Hashemi Gahruie, Ziaee, Eskandari, & Hosseini, 2017).

### 2.5 Determination of radical scavenging activity

The radical scavenging activity (RSA) was measured by 2,2‐diphenyl‐1‐picrylhydrazyl free radicals (DPPH°) according to the method described by Mazidi, Rezaei, Golmakani, Sharifan, and Rezazadeh (2012) and Mojtahed Zadeh Asl, Niakousari, Hashemi Gahruie, Saharkhiz, and Mousavi Khaneghah (2018) with some modifications. Briefly, 0.2 ml of kombucha samples was mixed with 0.8 ml methanol, and a range of volumes for this stock solution was prepared that ranged from 0.0125 to 0.2 ml/ml. Then, 0.5 ml of each diluted sample was mixed thoroughly with 1.5 ml of 0.1 mM DPPH° solution. The solution was incubated at room temperature for 1 hr in a dark place, and the reduction in DPPH° was measured by reading the absorbance at 517 nm. Samples without tea solutions served as the control. The IC_50_ values were defined as the concentration of an antioxidant which is required to reduce the initial DPPH concentration by 50%. The DPPH° inhibition activity was calculated according to the following equation:

(1) $${\text{DPPH}}^{\circ }\;\text{inhibition}\;\text{activity}\left(\%\right)=\left[\left(A_{\mathrm{control}}-A_{\mathrm{sample}}\right)/A_{\mathrm{control}}\right]\times 100$$

### 2.6 Total phenolic content

The total phenolic content (TPC) was measured by Folin–Ciocalteu based on the method described by Habibi, Golmakani, Mesbahi, Majzoobi, and Farahnaky (2015). Kombucha tea was then mixed with the 0.75 ml of Folin–Ciocalteu (10% w/w in distilled water) and was kept in the dark at ambient temperature for 10 min. Afterward, 0.75 ml of sodium carbonate at 0.2% was added to the solution, and the mixture was placed in a dark place for 1 hr. Absorptions were measured at 765 nm. The TPC values were determined via a calibration curve prepared with a series of gallic acid standards (25, 50, and 100 μg/ml). Results are expressed as mg of the gallic acid equivalent per mL of kombucha weight.

### 2.7 Total flavonoid content

The procedure described by Habibi et al. (2015) was pursued to determine total flavonoid content (TFC) of kombucha teas. Briefly, 0.5 ml of kombucha teas and 0.5 ml of methanol were mixed with 0.1 ml of 10% AlCl_3_, 0.1 ml of 1 M CH_3_CO_2_K, and 2.8 ml of distilled water. After incubation at ambient temperature for 30 min, the absorbance was measured at 415 nm. Different concentrations of Quercetin (6.25, 12.00 and 25.00 μg/ml methanol) were used in the plotting of the standard calibration curve. The TFC was expressed as mg of quercetin equivalent per g of fresh kombucha tea.

### 2.8 Minimum inhibitory concentration

The term “MIC” is defined as the minimum concentration of an antimicrobial agent that inhibits growth of microorganisms to the extent that no growth would be visibly observable in the culture medium. The broth microdilution method was applied to determine the MIC. Briefly, broth subcultures were prepared by inoculating one colony from each bacterium (*S. aureus*,* B. cereus, E. coli*, and *S. typhimurium*) grown overnight inside a 100‐ml flask containing 25 ml Mueller Hinton Broth (MHB) which was then incubated at 37 ± 2°C for 24 hr in a water bath shaker (Innova 3100, Brunswick Scientific, Edison, NJ, USA) at 160 rpm. Then, the concentration of each bacterium was adjusted to a final density of 1.5 × 10^6^ CFU/ml followed by harvesting the cells by centrifugation (MIKRO 120, Hettich, Tuttlingen, Germany) at 10,000 × *g* for 10 min. Then, these were suspended in a sterile saline solution (0.85% NaCl) and were used for inoculation by 96‐well microplates containing serial dilutions of the kombucha teas ranging from 0.125 to 1.000 μl. The micro‐plates were incubated at 37 ± 2°C for 24 hr. After incubation, bacterial growth was estimated by turbidity (Jacobsen et al., 2011).

### 2.9 Sensorial evaluation

The sensorial evaluation was performed by 12 semitrained panelists. Sensorial evaluation was based on a five‐point hedonic scale (5 = very good, 4 = good, 3 = middle, 2 = bad, and 1 = very bad). Sensorial evaluation parameters included flavor, odor, color, sourness, and overall acceptability.

### 2.10 Statistical analyses

All experiments were performed in triplicates. Analysis of variance (ANOVA) and the Duncan multiple range test were performed to determine significant differences (*p *<* *0.05) among the mean values (SAS ver. 9.1, 2002–2003 by SAS Institute Inc., Cary, NC, USA) (Hosseini et al., 2019).

## 3 RESULTS AND DISCUSSION

### 3.1 Part 1: Effects of medicinal plant type on kombucha samples

#### 3.1.1 Organic acids

Organic acid contents of flavored kombucha are shown in Table 1. Relevant results showed that acetic acid was the major acid in all kombucha samples. Cinnamon‐flavored kombucha had the highest of acetic acid content. Acetic acid significantly (*p *<* *0.05) increased during fermentation—so much so that the initial and final concentrations of acetic acid were 737.84–982.91 and 1,131.8–2,675.36 mg/L, respectively. Chen and Liu (2000) evaluated different treatments by collecting tea fungus samples from nine households scattered throughout Taiwan and investigated them on kombucha under a period of 60 days. They observed that the acetic acid concentration increased up to 8,000 mg/L at the end of the storage period. Jayabalan et al. (2007) studied changes in the organic acid contents of kombucha tea during fermentation. They observed that green tea had the highest amount of acetic acid (9,500 mg/L) on day 15 of fermentation. Glucuronic acid was the second most abundant acid in kombucha samples. This acid did not exist in the initial stage of fermentation, but reached a quantitative range of 839.06–1,158.2 mg/L at the end of fermentation. Chen and Liu (2000) evaluated different treatments on kombucha for 60 days. At the end of the fermentation process, the amount of glucuronic acids reached 39,000 mg/L. Glucuronic acid is considered as a key component in the bioactivity of kombucha tea with respect to its detoxifying action (Vina, Semjonovs, Linde, & Patetko, 2013). Yang et al. (2010) isolated the symbiotic bacteria *Gluconacetobacter sp*. A4 and also 10 species of lactic acid bacteria from kefir, and subsequently inoculated them to be incorporated into the kombucha. They observed that the highest glucuronic acid in black tea was 5,000 mg/L.

**Table 1:** Effect of different medicinal plant types and concentrations on major organic acid contents of flavored kombucha

Treatment	Fermentation time (day)	Organic acid (mg/L)						
		Acetic acid	D‐Glucuronic acid	Lactic acid	Citric acid	Oxalic acid	Tartaric acid	Malic acid
Cinnamon‐flavored kombucha	0	982.91 ± 67.12	ND	83.42 ± 9.52	ND	6.4 ± 0.78	ND	ND
	16	2,675.36 ± 239.01	1,158.2 ± 238.56	184.57 ± 11.67	471.58 ± 44.50	11.4 ± 0.94	ND	276 ± 23.13
Cardamom‐flavored kombucha	0	793.51 ± 59.13	ND	45.9 ± 7.54	ND	6.3 ± 0.34	ND	ND
	16	1,288.54 ± 98.65	118.92 ± 13.78	140.41 ± 11.54	254.57 ± 29.51	8.4 ± 0.67	31.4 ± 2.89	89 ± 11.27
Shirazi thyme‐flavored kombucha	0	737.84 ± 47.89	ND	22.99 ± 4.78	ND	8.8 ± 1.10	ND	ND
	16	1,131.8 ± 136.79	ND	90.81 ± 10.45	39.22 ± 4.45	24.8 ± 3.14	73.4 ± 9.32	312 ± 32.67
Kombucha tea	0	944.54 ± 81.12	ND	48.35 ± 3.21	ND	10.4 ± 0.96	ND	ND
	16	2,395.64 ± 189.12	839.06 ± 94.13	145.71 ± 17.76	268.41 ± 23.97	12.1 ± 2.23	ND	113 ± 9.73
Cinnamon (100%)	0	855.62 ± 67.11	34.47 ± 6.78	ND	ND	4.1 ± 0.23	ND	76 ± 8.42
	16	2,755.75 ± 98.55	1,106.32 ± 98.34	231.47 ± 28.34	ND	13.1 ± 2.08	ND	256 ± 31.34
Cinnamon (75%)	0	1,008.56 ± 82.18	ND	16.09 ± 1.59	ND	4.5 ± 0.22	ND	82 ± 9.56
	16	2,556.04 ± 79.22	1,114.05 ± 179.13	269.83 ± 39.54	ND	16.6 ± 2.19	16.8 ± 2.68	224 ± 27.38
Cinnamon (50%)	0	982.91 ± 56.19	ND	83.42 ± 11.79	ND	6.4 ± 0.39	ND	ND
	16	2,675.36 ± 267.09	1,158.2 ± 232.89	184.57 ± 22.07	471.58 ± 56.43	11.4 ± 1.67	ND	276 ± 31.11
Cinnamon (25%)	0	892.37 ± 45.44	76.28 ± 11.78	ND	ND	5.2 ± 0.65	ND	98 ± 11.97
	16	2,540.36 ± 145.15	839.06 ± 78.91	150.89 ± 11.65	244.63 ± 43.12	12.2 ± 0.89	ND	182 ± 14.79

*Note*

ND: not detected.

Citric acid did not exist in the initial stage of fermentation but then appeared to be produced toward the end of the fermentation process and was present at concentrations of 39.22–471.58 mg/L. The lactic acid concentration significantly (*p *<* *0.05) increased during fermentation. Its concentration measured 22.09–83.42 mg/L at the beginning, but measured 90.81–184.57 mg/L at the end of the fermentation process. Malbaša, Lončar, and Djurić (2008) used molasses as carbon sources for kombucha fermentation. They reported that the L‐lactic content significantly (*p *<* *0.05) increased during kombucha fermentation. Malic and oxalic acid concentrations, respectively, measured 89–276 and 8.4–24.8 mg/L at the end of fermentation in kombucha samples.

The concentration of all organic acids rose significantly (*p *<* *0.05) during the fermentation process of both flavored kombucha and the control. Cinnamon‐flavored kombucha had the highest acetic acid, D‐glucuronic acid, lactic acid, and citric acid contents while Shirazi thyme‐flavored kombucha had the highest oxalic acid (24.8 mg/L) and malic acid contents (312 mg/L), at the end of the fermentation process.

#### 3.1.2 pH

**Figure 1:**
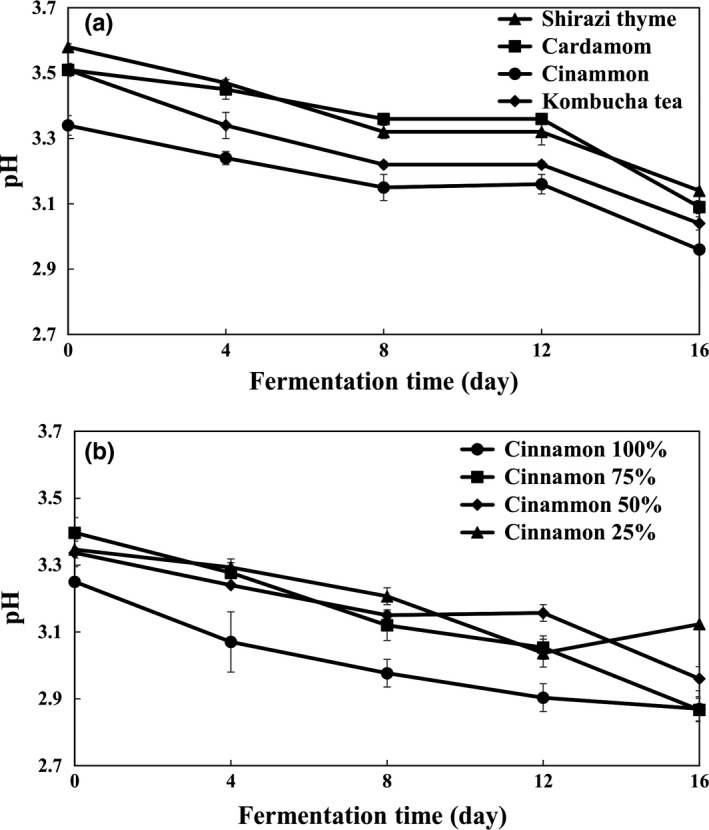
Effect of medicinal plants (a) type and (b) concentration on pH of flavored kombucha

Comparisons between the pH of flavored kombucha samples are shown in Figure 1. The pH of kombucha samples decreased significantly (*p *<* *0.05) during fermentation, mainly due to the increase in organic acid contents. However, at the beginning of fermentation, no significant (*p *<* *0.05) differences were observed in the pH of kombucha samples in comparison with kombucha tea, whereas the cinnamon‐flavored kombucha exhibited the lowest pH (2.96) at the end of fermentation. The lowest pH observed in cinnamon‐flavored kombucha can be explained by the presence of higher amounts of acetic, citric, malic, lactic, and glucuronic acids. Kombucha samples ultimately staged a gradual decrease in pH which is due to their buffering properties. In this regard, our results are in agreement with those of Chen and Liu (2000) and Sreeramulu, Zhu, and Knol (2000).

#### 3.1.3 TPCs

**Figure 2:**
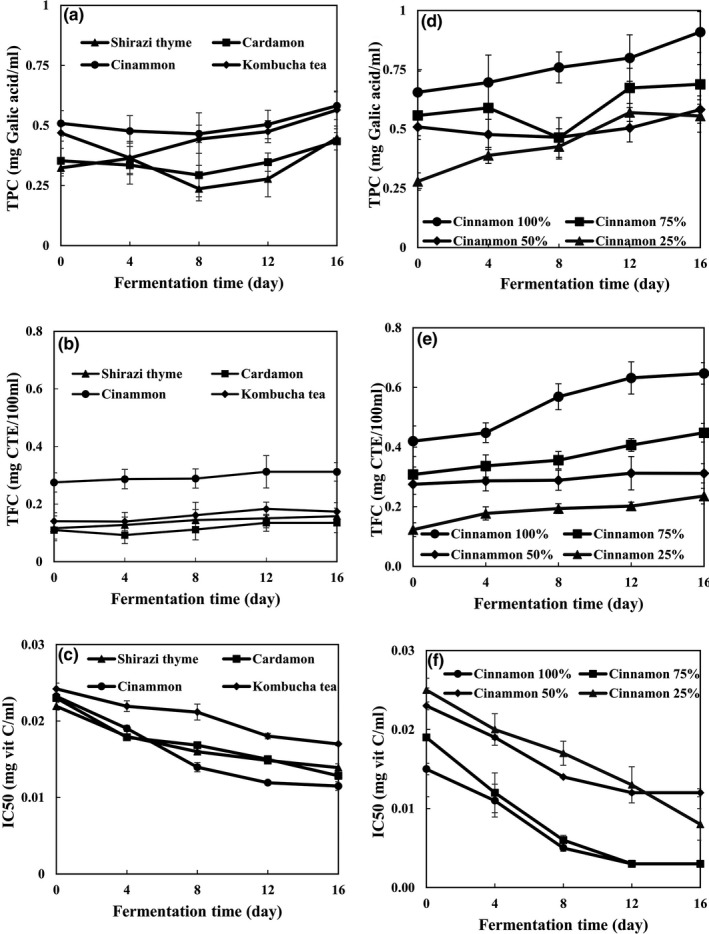
Effect of medicinal plant types (a–c) and medicinal plant concentrations (d–f) on total phenolic content (TPC), total flavonoid content (TFC), and the IC_50_ values of flavored kombucha during fermentation time

The TPCs of flavored kombucha samples are illustrated in Figure 2. The TPCs in all samples increased significantly (*p *<* *0.05) during fermentation. Although at the beginning of fermentation, the TPC values remained almost constant, they increased significantly (*p *<* *0.05) at the end of fermentation. TPCs of samples decreased during storage due to the acidic nature of the kombucha drink and its enzymes (Jayabalan et al., 2007). Previous studies by Hoon, Choo, Watawana, Jayawardena, and Waisundara (2014) reported increases in the TPCs of kombucha samples at the beginning of fermentation.

Total phenolic contents of cinnamon‐flavored kombucha were measured and observed to be comprised of 0.582 mg galic acid/ml. The kombucha tea, however, contained 0.565 mg galic acid/ml. Lower amounts of TPCs were observed in the cardamom‐flavored kombucha and Shirazi thyme‐flavored kombucha at the end of fermentation. Shan, Cai, Sun, and Corke (2005) in a relevant research studied 26 plant species contextually and reported that the highest TPCs were found in the cinnamon and oregano.

#### 3.1.4 TFCs

The TFCs of flavored kombucha are shown in Figure 2. The TFCs of kombucha samples significantly (*p *<* *0.05) increased during fermentation. Release of TFCs (catechins) from the acid‐sensitive microbial cells led to high concentrations of TFCs (epicatechin isomers) observed on the 12th day of fermentation. The rise in TFC concentrations, as observed on the 12th day, could be explained by certain biotransformations leading to the enzymatic production of EGC and EC. Previous studies show that theaflavin and thearubigen can remain to be stable structures when compared with epicatechin isomers during kombucha fermentation (Jayabalan et al., 2007).

The cinnamon‐flavored kombucha (0.312 mg CTE/100 ml) had the highest TFCs, while the cardamom‐flavored kombucha (0.135 mg CTE/ml) had the lowest TFCs at the end of fermentation.

#### 3.1.5 RSA

The IC_50_ values of flavored kombucha samples are shown in Figure 2. However, IC_50_ values of all samples decreased significantly (*p *<* *0.05) during fermentation, which denotes increasing antioxidant activity. The increase in antioxidant activity of kombucha samples is mainly due to the low molecular weights of the produced polyphenols and is also accompanied by various changes to the structures of polyphenols (Jayabalan, Malbaša, Lončar, Vitas, & Sathishkumar, 2014). In another experiment, kombucha tea was produced by the integration of green tea, black tea, and waste tea. Results showed that the antioxidant activity rises in response to free radicals, and also in response to superoxide radicals with reducing powers, alongside the involvement of antilipid peroxides in the course of fermentation (Jayabalan et al., 2007).

Kombucha tea exhibited the highest IC_50_ value (0.017 mg vit C/ml) while cinnamon‐flavored kombucha manifested the lowest IC_50_ value (0.012 mg vit C/ml). Furthermore, Velićanski et al. (2014) used lemon balm and black tea for fermentation of kombucha samples. They observed that the antioxidant activity of fermented kombucha black tea was relatively higher than the fermented kombucha by lemon balm. This result, however, stands in contrast with our results.

#### 3.1.6 MIC

MIC values of flavored kombucha samples are shown in Table 2. Antibacterial activity of all medicinal plants CIN, CAR, and SHT was assayed and compared to the kombucha tea, against four pathogenic bacteria, namely, *S. aureus*,* B. cereus, E. coli* O157:H7, and *S. typhimurium*.

**Table 2:** Effect of medicinal plant types and concentrations on minimum inhibitory concentration (mg/L) of flavored kombucha

Treatments	Microorganisms	Storage time (day)				
		0	4	8	12	16
*Staphylococcus aureus*	Cinnamon‐flavored kombucha	5,833 ± 2,020	4,666 ± 2,020	3,500 ± 0	3,500 ± 0	3,500 ± 0
	Cardamom‐flavored kombucha	7,000 ± 0	5,833 ± 2,020	4,666 ± 2,020	3,500 ± 0	3,500 ± 0
	Shirazi thyme‐flavored kombucha	5,833 ± 2,020	4,666 ± 2,020	3,500 ± 0	3,500 ± 0	3,500 ± 0
	Kombucha tea	7,000 ± 0	5,833 ± 2,020	3,500 ± 0	5,833 ± 2,020	3,500 ± 0
*Bacillus cereus*	Cinnamon‐flavored kombucha	3,500 ± 0	3,500 ± 0	3,500 ± 0	3,500 ± 0	3,500 ± 0
	Cardamom‐flavored kombucha	4,666 ± 2,020	4,666 ± 2,020	3,500 ± 0	4,666 ± 2,020	2,916 ± 1,010
	Shirazi thyme‐flavored kombucha	3,500 ± 0	2,916 ± 1,010	4,666 ± 2,020	3,500 ± 0	3,500 ± 0
	Kombucha tea	3,500 ± 0	3,500 ± 0	3,500 ± 0	4,666 ± 2,020	3,500 ± 0
*Escherichia coli*	Cinnamon‐flavored kombucha	4,666 ± 2,020	2,916 ± 1,010	3,500 ± 0	1,750 ± 0	1,750 ± 0
	Cardamom‐flavored kombucha	5,833 ± 2,020	3,500 ± 0	3,500 ± 0	3,500 ± 0	3,500 ± 0
	Shirazi thyme‐flavored kombucha	3,500 ± 0	3,500 ± 0	3,500 ± 0	3,500 ± 0	3,500 ± 0
	Kombucha tea	2,916 ± 1,010	2,916 ± 1,010	3,500 ± 0	3,500 ± 0	2,916 ± 1,010
*Salmonella typhimurium*	Cinnamon‐flavored kombucha	4,666 ± 2,020	3,500 ± 0	3,500 ± 0	2,916 ± 1,010	2,333 ± 1,010
	Cardamom‐flavored kombucha	7,000 ± 0	3,500 ± 0	3,500 ± 0	3,500 ± 0	3,500 ± 0
	Shirazi thyme‐flavored kombucha	3,500 ± 0	3,500 ± 0	3,500 ± 0	3,500 ± 0	2,916 ± 1,010
	Kombucha tea	3,500 ± 0	3,500 ± 0	3,500 ± 0	3,500 ± 0	3,500 ± 0
*S. aureus*	Cinnamon 100%	7,000 ± 0	3,500 ± 0	2,916 ± 1,010	2,916 ± 1,010	2,916 ± 1,010
	Cinnamon 75%	7,000 ± 0	4,666 ± 2,020	2,916 ± 1,010	4,666 ± 2,020	3,500 ± 0
	Cinnamon 50%	5,833 ± 2,020	4,666 ± 2,020	3,500 ± 0	3,500 ± 0	3,500 ± 0
	Cinnamon 25%	7,000 ± 0	4,666 ± 2,020	5,833 ± 2,020	7,000 ± 0	4,666 ± 2,020
*B. cereus*	Cinnamon 100%	4,666 ± 2,020	3,500 ± 0	1,458 ± 505	2,916 ± 1,010	2,333 ± 1,010
	Cinnamon 75%	5,833 ± 2,020	4,666 ± 2,020	2,916 ± 1,010	3,500 ± 0	2,916 ± 1,010
	Cinnamon 50%	3,500 ± 0	3,500 ± 0	3,500 ± 0	3,500 ± 0	3,500 ± 0
	Cinnamon 25%	7,000 ± 0	4,666 ± 2,020	4,666 ± 2,020	4,666 ± 2,020	4,666 ± 2,020
*E. coli*	Cinnamon 100%	4,666 ± 2,020	3,500 ± 0	2,916 ± 1,010	2,916 ± 1,010	1,750 ± 0
	Cinnamon 75%	5,833 ± 2,020	3,500 ± 0	4,666 ± 2,020	2,916 ± 1,010	3,500 ± 0
	Cinnamon 50%	4,666 ± 2,020	2,916 ± 1,010	3,500 ± 0	1,750 ± 0	1,750 ± 0
	Cinnamon 25%	5,833 ± 2,020	5,833 ± 2,020	5,833 ± 2,020	5,833 ± 2,020	4,666 ± 2,020
*S. typhimurium*	Cinnamon 100%	5,833 ± 2,020	2,916 ± 1,010	3,500 ± 0	2,333 ± 1,010	2,333 ± 1,010
	Cinnamon 75%	7,000 ± 0	4,666 ± 2,020	4,666 ± 2,020	4,666 ± 2,020	3,500 ± 0
	Cinnamon 50%	4,666 ± 2,020	3,500 ± 0	3,500 ± 0	2,916 ± 1,010	2,333 ± 1,010
	Cinnamon 25%	7,000 ± 0	4,666 ± 2,020	7,000 ± 0	5,833 ± 2,020	5,833 ± 2,020

Although antibacterial activities of all flavored kombucha samples significantly increased (*p *<* *0.05) during fermentation period, but no significant (*p *<* *0.05) differences were observed between the MIC values of samples measured on the 4th day and the 16th day. The components comprising the essential oil obtained by the distillation of cinnamon demonstrate that these components have both non‐volatile compounds and volatile compounds, though considerations must be made with respect to the highest antibacterial activities and the precursors that lead to such outcomes, as compared with the rest of treatments (Burt, 2004).

At the end of the fermentation process, the result showed that cinnamon‐flavored kombucha has significant (*p *<* *0.05) inhibitory effects on *E. coli*,* S. typhimurium,* and *S. aureus*. The least inhibitory effect was observed in the cardamom‐flavored kombucha. Against the *B. cereus* bacteria, no significant (*p *<* *0.05) difference of inhibition was observed among the samples containing the various medicinal plants. In a relevant study, Valero and Salmeron (2003) reported the inhibitory effects of 11 essential oils, namely, nutmeg, mint, clove, oregano, cinnamon, sassafras, sage, thyme, and rosemary on *B. cereus*. Furthermore, Shan, Cai, Brooks, and Corke (2007) reported the effect of the cinnamon stick and its extract compounds on pathogenic bacteria such as *B. cereus*,* Listeria monocytogenes*,* S. aureus*,* E. coli*, and *Salmonella anatum* by measuring the MIC.

By comparing the effects of different medicinal plants on Gram‐positive and Gram‐negative pathogens, results show that among the Gram‐positive bacteria, *B. cereus* was most susceptible, while among the Gram‐negative bacteria, *E. coli* was the most susceptible species. Antibacterial activities of kombucha samples occurred against both Gram‐positive and Gram‐negative pathogens. The efficiency of antibacterial activities can depend on organic acids, especially acetic acid and TFCs (Sreeramulu et al., 2000). A study by Greenwalt, Ledford, and Steinkraus (1998) evaluated the antibacterial activities of kombucha tea against *Agrobacterium tumefaciens*,* B. cereus*,* Salmonella choleraesuis*,* S. typhimurium*,* S. aureus*,* E. coli*, and *Candida albicans*, whereby the growths of all these bacterial species were considerably inhibited, except for *C. albicans*.

#### 3.1.7 Sensorial evaluation

Sensorial evaluations of flavored kombucha samples resulted in varied outcomes (Table 3). Results show that the cinnamon‐flavored kombucha had the highest scores on flavor, odor, pleasantness, acidity, and color, in comparison with other samples. The cinnamon used herein, as a coffee supplement, affects not only the flavor and aroma of the kombucha, but also its antioxidant activities, since cinnamon is a source of bioactive compounds such as cinnamic acid, eugenol, and coumarin (Shan et al., 2005).

**Table 3:** Effect of medicinal plant types on sensorial evaluation the flavored kombucha samples

Treatments	Sensorial characteristic				
	Odor	Flavor	Sourness	Color	Overall acceptability
Cinnamon‐flavored kombucha	3.87 ± 0.83	4.00 ± 0.75	3.87 ± 0.64	3.75 ± 0.70	3.75 ± 0.70
Cardamom‐flavored kombucha	3.62 ± 0.74	3.75 ± 0.75	3.37 ± 0.51	3.62 ± 0.74	3.37 ± 0.51
Shirazi thyme‐flavored kombucha	3.12 ± 0.64	2.75 ± 0.70	2.87 ± 0.83	2.87 ± 0.64	2.62 ± 0.74
Kombucha tea	3.37 ± 0.51	3.50 ± 0.51	3.75 ± 0.70	3.50 ± 0.53	3.50 ± 0.53

### 3.2 Part 2: Effects of cinnamon concentration on kombucha samples

Based on the sensorial evaluation results, concerning the first part of the experiment, the cinnamon was selected as the most suitable medicinal plant with regard to the optimality of its antimicrobial and antioxidant activities.

#### 3.2.1 Organic acids

The effect of different cinnamon concentrations (25%, 50%, 75%, and 100%) on organic acid content of flavored kombucha samples is shown in Table 1. The highest amounts of organic acids resulted from CIN 100% while the lowest related to the CIN 25%. By increasing the cinnamon concentrations, acetic acid, D‐glucuronic acid, lactic acid, oxalic acid, and malic acid contents also increased. Accordingly, the highest organic acid contents were acetic acid, D‐glucuronic acid, lactic acid, and malic acid, among which the amount of acetic acid was the highest. Acetic acid concentration decreased, parallel to the decrease in the concentration of CIN. Where CIN was applied at 25%, the acetic acid concentration became 2,540.36 mg/L, whereas CIN at its 100% concentration caused acetic acid to become 2,755.75 mg/L. Furthermore, D‐glucuronic acid was the second most abundant acidic component in flavored kombucha samples and its concentration increased by increasing the cinnamon concentration from 25% (839.06 mg/L) to 100% (1,106.32 mg/L). Citric acid and lactic acid concentrations were, respectively, 244.63 and 150.89 mg/L, occurring by the CIN 25%, while by the CIN 100%, the respective concentration of citric acid was not detected, but showed to contain 231.47 mg/L of lactic acid. The malic acid and oxalic acid concentrations were 256.0 and 13.1 mg/L, respectively, by the CIN 100% were 256.0 and 13.1 mg/L, respectively, whereas tartaric acid was not detected.

#### 3.2.2 pH

The effect of different cinnamon concentrations (25%, 50%, 75%, and 100%) on pH of flavored kombucha samples is shown in Figure 1. The minimum value of pH was 2.87 which resulted from the CIN 100%. A consistent correlation was also observed between the increasing amounts of organic acid contents and the reduction in pH value.

#### 3.2.3 TPC

The effect of different cinnamon concentrations on TPCs of flavored kombucha samples is shown in Figure 2. The results represent significant (*p *<* *0.05) trends of increase in the TPCs along with the parallel increase in cinnamon concentrations during fermentation. According to the results, the highest TPCs were found in relation to CIN 100% which yielded 0.555 mg GAE/ml.

#### 3.2.4 TFC

The effect of different cinnamon concentrations on TFC of flavored kombucha samples is shown in Figure 2. Different concentrations of cinnamon established varied ranges of positive effects on the TFC values. It is evident that by increasing the cinnamon concentration, the TFC value significantly (*p *<* *0.05) increased. Maximum TFC at the end of the fermentation process was 0.647 mg CTE/ml which occurred by the CIN concentration of 100%.

#### 3.2.5 RSAs

The effect of different cinnamon concentrations on RSAs of flavored kombucha samples is shown in Figure 2. Higher concentrations of cinnamon had more positive effects on RSA values. The results in Figure 2 signify that the maximum magnitude of RSA results from cinnamon concentration of 100%. However, increasing the cinnamon concentration affected the IC_50_ too, whereby increasing the RSA values caused significant (*p *<* *0.05) reductions in the IC_50_ values. The highest IC_50_ value corresponded to the CIN 50% (0.012 mg vit C/ml). But on the other hand, the CIN concentration of 100% had the lowest IC_50_ value of 3 mg vitamin C/L. The cinnamon used herein, as a coffee supplement, affects antioxidant activities because the cinnamon is a good source of bioactive compounds such as cinnamaldehyde, eugenol, and coumarin (Shan et al., 2005). The effect of cinnamon extract serving as a free radical scavenger has been previously reported by Roussel, Hininger, Benaraba, Ziegenfuss, and Anderson (2009).

A consistent negative correlation was observed between the TPCs and RSAs (0.704) and also between the TFC and RSAs (0.726). By higher amounts of the TFC and TPC, the amounts of RSA values decreased significantly (*p *<* *0.05).

#### 3.2.6 MIC

The effect of different cinnamon concentrations on MIC of flavored kombucha samples is shown in Table 2. By increasing the cinnamon concentration, the MIC decreased significantly (*p *<* *0.05) during fermentation. Therefore, different cinnamon concentrations can favorably affect the MIC. The highest inhibitory activity was caused by the cinnamon concentration of 100%.

When comparing the Gram‐positive (*S. aureus*) and Gram‐negative (*E. coli*) pathogens, it can be inferred that the Gram negatives are more susceptible. From a molecular perspective, the cinnamon components destroy the cytoplasmic membrane of Gram‐positive and Gram‐negative bacteria and induce the depletion of intracellular ATP concentrations. The aqueous extract of cinnamon is also known to have antibacterial effects against *Helicobacter pylori* (Nuryastuti et al., 2009). Furthermore, the MIC values decreased significantly (*p *<* *0.05) as the pH decreased.

## 4 CONCLUSION

This study serves as a contribution to the hypothesis that medicinal plants can further promote the kombucha in being identified as a functional beverage product. By evaluating the effects of three medicinal plant types and cinnamon concentrations on the physicochemical, antioxidant, antimicrobial, and sensorial properties of flavored kombucha samples, this study revealed that, among the three medicinal plants used herein, the fermented CIN exhibited the highest antioxidant, antimicrobial, and sensorial properties at the end of the fermentation process. In the second part of the experiment, the effects of various cinnamon concentrations were studied. Results show that increasing the cinnamon concentration leads to significant increases in the amounts of organic acids and antioxidants, besides higher antimicrobial activities in the flavored kombucha. These findings can be deemed very promising when considering the development of such newly advanced products, not only because of their high level of agreeableness in terms of flavor and odor, but also because of their improved bioactivity and health benefits compared to traditional kombucha tea.

## CONFLICT OF INTEREST

The authors declare no conflict of interest.
